# Mental Health and Quality of Life in Patients with Untreated Unruptured Intracranial Aneurysms: A Systematic Review and Meta-Analysis of 417,152 Patients with Trial Sequential Analysis

**DOI:** 10.3390/brainsci15070764

**Published:** 2025-07-18

**Authors:** Plamen Penchev, Kiril Ivanov, Daniela Milanova-Ilieva, Lyubomir Gaydarski, Kiril Kostov, Nikola Boyadzhiev, Petar-Preslav Petrov, Patrice Mehandzhiev, Remzi Hyusein, Vladislav Velchev, Ilko Ilyov, Valentin Kuzmanov, Gergana Dzhikova, Desislava Dobreva, Liliana Toptchiyska, Vasilena Dimitrova, Victoria Petrova, Svetoslav Yorov, Pavel Stanchev, Martin Gyulbaharov, Noor Husain, Nikolai Ramadanov

**Affiliations:** 1 Faculty of Medicine, Medical University of Plovdiv, 4002 Plovdiv, Bulgaria; 2 Department of Pediatrics, St. George’s University Hospital, 4000 Plovdiv, Bulgaria; 3 Department of Anatomy, Histology and Embryology, Medical University of Sofia, 1431 Sofia, Bulgaria; 4 Department of Anatomy, Histology and Embryology, Medical University of Plovdiv, 4002 Plovdiv, Bulgaria; 5 Neurosurgery Clinic, University Hospital “Pirogov”, 1606 Sofia, Bulgaria; 6 Faculty of Medicine, Medical University of Sofia, 1431 Sofia, Bulgaria; 7 Faculty of Medicine, Sofia University, 1504 Sofia, Bulgaria; 8 Faculty of Medicine, Medical University of Pleven, 5800 Pleven, Bulgaria; 9 Eye Clinic Den, 1000 Sofia, Bulgaria; 10 Department of Anesthesiology, Klinik Hietzing, 1130 Wien, Austria; 11 Clinic of Endocrinology and Metabolic Diseases, St. George’s University Hospital, Medical University of Plovdiv, 4002 Plovdiv, Bulgaria; 12 Department of Anesthesiology, Asklepios Clinic, 23843 Bad Oldesloe, Germany; 13 Department of Pharmacology, Indira Gandhi Institute of Medical Sciences, Patna 800014, India; 14 Center of Orthopaedics and Traumatology, Brandenburg Medical School, University Hospital Brandenburg, 14770 Brandenburg an der Havel, Germany; 15 Faculty of Health Science Brandenburg, Brandenburg Medical School Theodor Fontane, 14770 Brandenburg an der Havel, Germany

**Keywords:** unruptured intracranial aneurysm (UIA), untreated UIAs, depression, anxiety, mental health, quality of life (QoL)

## Abstract

**Introduction:** Unruptured intracranial aneurysms (UIAs) can induce psychological stress, leading to anxiety, depression, and impaired quality of life (QoL). Most studies on this topic are limited by small sample sizes, cross-sectional designs, and a focus on treated rather than untreated cases, leaving a gap in the literature. We aimed to conduct a systematic review and meta-analysis to evaluate mental health and QoL outcomes in patients with untreated UIAs. **Methods:** A systematic search was conducted up to 30 November 2024 using PubMed, Scopus, and Cochrane Central for studies comparing patients with untreated UIAs to a control group. The outcomes of interest included anxiety, depression, and QoL. Statistical analysis was performed using RevMan 5.1.7 and R 4.3.1. Heterogeneity was assessed using I^2^ statistics and the Cochrane Q test. Risk ratios (RR) and standardized mean differences (SMD) were computed using a frequentist random-effects model. **Results:** We included five studies with 417,152 patients, of whom 85,668 (20.53%) had untreated UIAs. In the pooled analysis, patients with untreated UIAs had significantly higher anxiety levels (SMD 0.66; 95% CI [0.16; 1.17]; *p* = 0.01; I^2^ = 76%) and lower QoL (SMD −0.82; 95% CI [−1.12; −0.53]; *p* = 0.01; I^2^ = 56%) compared to the control group However, no statistically significant differences were found in depression (RR 0.94; 95% CI [0.52; 1.72]; *p* = 0.84; I^2^ = 88%) between groups. **Conclusions:** This meta-analysis indicates a potential association between untreated UIAs and increased anxiety levels and reduced QoL. Regarding depression, no significant differences were observed between groups.

## 1. Introduction

Unruptured intracranial aneurysms (UIAs) are acquired vascular abnormalities characterized by the dilation of arterial walls in the brain. These aneurysms are relatively common, affecting approximately 1–2% of the general population, with most located in the anterior circulation of the circle of Willis [1]. The primary risk associated with UIAs is rupture, which can lead to subarachnoid hemorrhage (SAH)—a condition with mortality rates of 32–67% and significant long-term disability among survivors [2]. Recent advances in medical imaging have led to increased detection of UIAs, which are often identified incidentally in asymptomatic individuals. Despite this, the optimal management strategies for UIAs remain a topic of ongoing debate.

While small UIAs (<7 mm) in the anterior circulation carry a low risk of rupture over five years, larger or morphologically complex aneurysms may warrant intervention, such as surgical clipping or endovascular coiling [3]. However, these interventions have significant risks, including procedural morbidity and mortality. Diagnosing a UIA can induce considerable psychological stress, with patients often fearing life-threatening complications. Many describe living with an untreated UIA as having a “ticking time bomb” in their heads, resulting in a substantial psychological burden [4]. Consequently, patients frequently opt for conservative management with regular imaging follow-ups, which may exacerbate psychological strain and contribute to psychiatric disorders such as anxiety and depression, as well as impaired quality of life (QoL).

Most existing studies on UIAs are limited by small sample sizes, cross-sectional designs, and a focus on treated rather than untreated cases, reducing their findings’ generalizability [5,6,7,8,9,10,11,12,13,14,15,16,17,18]. A previous meta-analysis has primarily addressed the prevalence and clinical management of depression and anxiety in patients with UIAs. However, it has not adequately examined the impact of UIAs on QoL [19]. Furthermore, many studies included in this meta-analysis involved treated patients, not addressing the knowledge gap regarding the mental health and QoL outcomes for those with untreated UIAs.

Given the lack of robust evidence so far, we conducted a systematic review and meta-analysis to evaluate the association between untreated UIAs and mental health outcomes, including anxiety, depression, and QoL. Our study synthesized data from multiple studies to provide a comprehensive understanding of the psychological burden associated with untreated UIAs. Unlike previous studies, such as Ignacio et al. [19], which reported the prevalence of anxiety and depression without clearly distinguishing between treated and untreated patients, our meta-analysis focuses exclusively on patients with untreated UIA. In addition, we examined QoL as a central outcome—a dimension often overlooked in previous reviews. Furthermore, we uniquely applied Trial Sequential Analysis (TSA) to strengthen our conclusions and reduce the risk of type I error. This meta-analysis aimed to evaluate mental health and QoL outcomes in patients with untreated UIAs informing future clinical guidelines and highlight the importance of addressing both the mental and physical health needs of patients with untreated UIAs.

## 2. Methods

### 2.1. Eligibility Criteria

This systematic review and meta-analysis followed the Cochrane Handbook for Systematic Reviews of Interventions and the Preferred Reporting Items for Systematic Reviews and Meta-Analysis Statement [20,21]. This meta-analysis did not require Institutional Review Board approval because it used data from previously published and publicly available articles. Studies that met all the following criteria were included in the meta-analysis: (1) observational studies (case-control, cohort, and cross-sectional), (2) studies with patients who have untreated unruptured intracranial aneurysms, (3) studies that report at least one of the following outcomes: anxiety, depression, QoL assessed using validated measurement tools, and (4) studies that include as a control group patients who underwent treatment, patients with ruptured aneurysms, patients with other diseases, and healthy individuals. Studies were excluded if they met one of the following criteria: (1) included only patients with ruptured and/or treated aneurysms, (2) did not report one of the specified outcomes (anxiety, depression, and QoL), (3) different study designs (case reports), (4) lacked an appropriate control group, and (5) overlapping population. This systematic review and meta-analysis were registered with the International Prospective Register of Systematic Reviews (PROSPERO) under the ID “CRD42024600943”. The PRISMA checklist can be found in the supplementary material.

### 2.2. Search Strategy and Data Extraction

We systematically searched PubMed, Scopus, and the Cochrane Central from inception to 30 November 2024 using the following search strategy: (“unruptured intracranial aneurysms” OR UIA) AND (untreated OR “conservative management” OR “natural history”). Restrictions were applied to only English-language articles. Gray literature was excluded. We manually searched the references of all included studies to identify any additional studies. Two authors (P.P. and K.I.) independently extracted data using predefined search criteria, quality assessment methods, and Rayyan software (https://new.rayyan.ai accessed on 31 October 2024) [22]. Any disagreements between these authors were resolved through consensus.

### 2.3. Endpoints and Subgroup Analyses

The meta-analysis included anxiety, depression, and QoL endpoints. Additionally, we conducted a subgroup analysis for each endpoint based on the risk of bias assessment.

### 2.4. Quality Assessment

The risk of bias was assessed using the Cochrane Collaboration’s tool for assessing the risk of bias in non-randomized studies of interventions (ROBINS-I) [23]. The ROBINS-I tool categorizes the risk of bias as low, moderate, serious, or critical. Two authors (P.P. and N.H.) independently performed the assessments, resolving disagreements through consensus. Publication bias was evaluated using funnel-plot analysis, plotting individual study weights against point estimates. Following Cochrane guidelines, the Egger test was not performed because fewer than 10 studies were included in the meta-analysis [20].

### 2.5. Statistical Analysis

Risk ratios (RRs) with 95% confidence intervals (CIs) were computed to compare effects for binary endpoints using the Mantel–Haenszel method [24,25]. For continuous outcomes, means and standard deviations were extracted, and comparisons between groups were made using a weighted standardized mean difference (SMD). A random-effects model was applied to all outcomes to account for demographic and methodological variability. Heterogeneity was assessed using the I^2^ statistic and Cochran’s Q test. Two-sided *p*-values < 0.05 were regarded statistically significant. The restricted maximum-likelihood estimator random-effects model was used for continuous data, and the Hartung–Knapp random-effects model was applied for binary data to account for heterogeneity and small sample sizes [26,27]. To minimize the risk of selection bias, subgroup analyses were performed based on the risk of bias assessment. Leave-one-out (LOO) sensitivity analyses were also conducted to assess the robustness of the findings. A Baujat plot was generated to identify studies that contribute most to heterogeneity and their influence on the overall meta-analysis results. This diagnostic tool visually represents the balance between a study’s contribution to heterogeneity (x-axis) and its weight in the meta-analysis (y-axis), aiding in the interpretation of outliers or highly influential studies. Statistical analyses were performed using R software version 4.3.1 [28] with the packages “metafor” and “meta” and Review Manager 5.4.1 (Nordic Cochrane Centre, The Cochrane Collaboration, Copenhagen, Denmark) [29].

### 2.6. Trial Sequential Analysis (TSA)

To assess the robustness of the meta-analysis and control for type I and type II errors due to sparse data or repeated significance testing, Trial Sequential Analysis (TSA) was performed. TSA was conducted using Trial Sequential Analysis Viewer version 0.9.5.10 Beta with a two-sided significance level of 5% and a power of 80% [30]. The required information size (RIS) was calculated based on an anticipated relative risk reduction (RRR) of [20%], a control event rate (CER) of [18%], and heterogeneity correction using a random-effects model. Adjusted cumulative Z-curves were plotted to evaluate whether the results reached the TSA monitoring boundaries for statistical significance or the RIS.

## 3. Results

### 3.1. Study Selection and Baseline Characteristics

**Figure 1:**
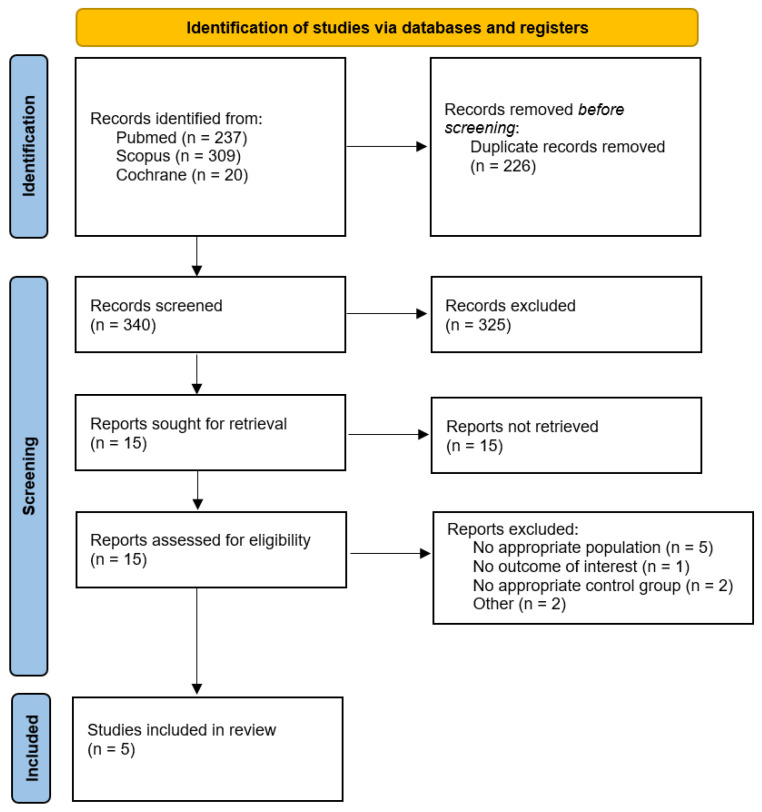
PRISMA flow chart and study selection.

The search strategy yielded a total of 566 results. After the removal of duplicate records and unrelated articles or abstracts, the remaining 15 studies were fully reviewed to determine whether they met the inclusion and exclusion criteria (Figure 1). Five studies were included, with a total of 417,152 patients [1,3,4,31,32]. Of those, 85,668 patients (20.53%) had untreated UIAs and were included in our analyses. The mean age of the population was ±56 years. The follow-up ranged from 5 to 10 years. Population characteristics are presented in Table 1.

### 3.2. Pooled Analyses of All Included Studies

#### 3.2.1. Anxiety

**Figure 2:**
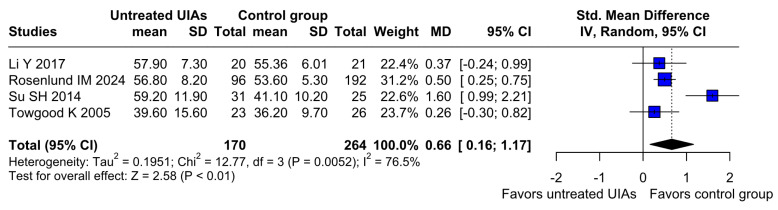
A statistically significant difference was found between patients with untreated UIAs and the control group, revealing that patients with untreated UIAs have higher anxiety levels [1,4,31,32].

**Figure 3:**
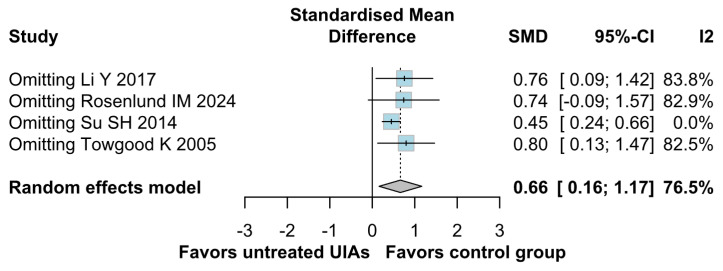
In terms of anxiety, the overall effect size remained consistent across all iterations, and the result remained significant in all cases. Heterogeneity remained high throughout all iterations [1,4,31,32].

**Figure 4:**
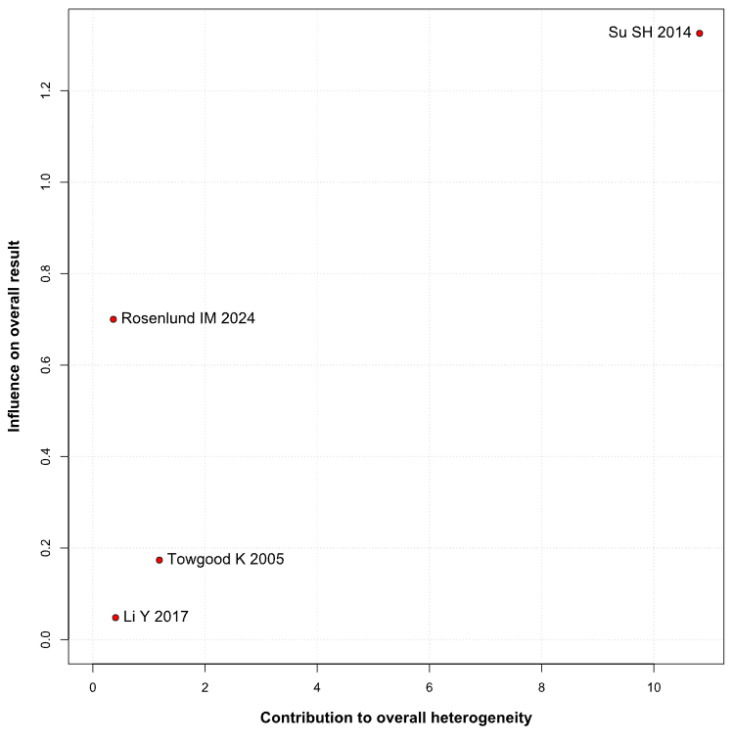
Baujat plot for anxiety, showing the contribution of individual studies to overall heterogeneity and their influence on the overall meta-analysis results [1,4,31,32].

Significantly higher anxiety levels were observed in patients with untreated UIAs (SMD 0.66; 95% CI [0.16; 1.17]; *p* = 0.01; I^2^ = 76%) compared to the control group (Figure 2). A LOO analysis was performed to test the robustness of our results. The overall effect size remained consistent across all iterations, and the result remained significant in all cases (SMD 0.66; 95% CI [0.16; 1.17]; *p* = 0.01; I^2^ = 76%) (Figure 3). This suggests that no single study has a disproportional influence on the overall outcome. The Baujat plot identified the study by Su SH 2014 et al. [32] as potentially influential, contributing substantially to the overall heterogeneity, and Rosenlund IM 2024 [31], contributing substantially to the overall result (Figure 4). High heterogeneity may be attributed to variations in measurement tools (e.g., HADS vs. BDI), population demographics, and study design differences across cohorts.

#### 3.2.2. Depression

**Figure 5:**
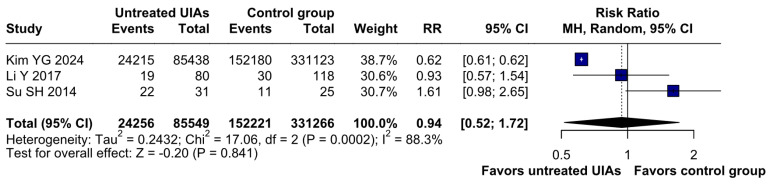
In terms of depression, the effect did not reach statistical significance [1,3,32].

**Figure 6:**
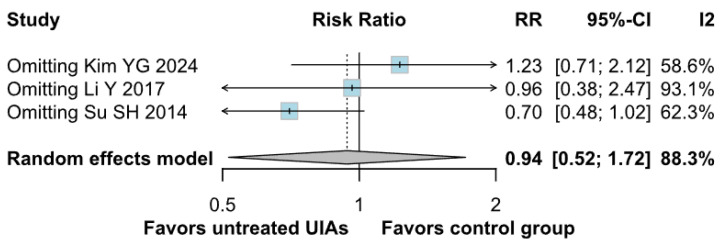
Regarding depression, the overall effect size remained consistent across all iterations and the result remained non-significant in all cases [1,3,32].

**Figure 7:**
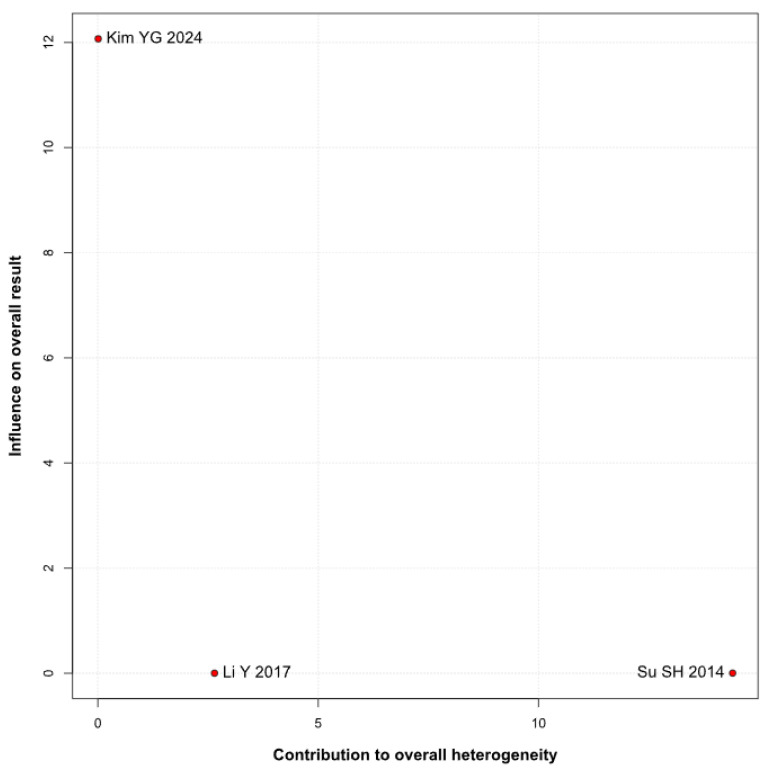
Baujat plot for depression, showing the contribution of individual studies to overall heterogeneity and their influence on the overall meta-analysis results [1,3,32].

There was no significant difference between the groups (RR 0.94; 95% CI [0.52; 1.72]; *p* = 0.84; I^2^ = 88%) (Figure 5). A LOO analysis was performed to test the robustness of our results. The overall effect size remained consistent across all iterations, and the findings did not differ significantly (RR 0.94; 95% CI [0.52; 1.72]; *p* = 0.84; I^2^ = 88%) (Figure 6). This suggests that no single study has a disproportional influence on the overall outcome. The Baujat plot identified the study by Su SH 2014 et al. [32] as potentially influential, contributing substantially to the overall heterogeneity, and Kim YG 2024 [3], contributing substantially to the overall result (Figure 7). High heterogeneity may be attributed to variations in measurement tools (e.g., HADS vs. BDI), population demographics, and study design differences across cohorts.

#### 3.2.3. QoL

**Figure 8:**
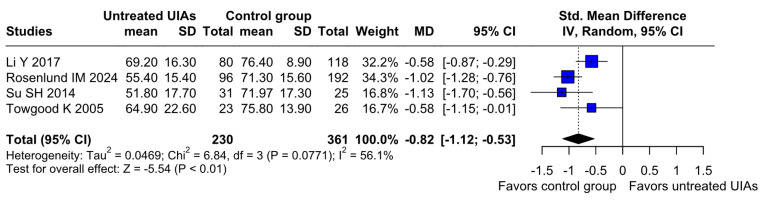
A statistically significant difference was found between patients with untreated UIAs and the control group, revealing that patients with untreated UIAs have lower QoL [1,4,31,32].

**Figure 9:**
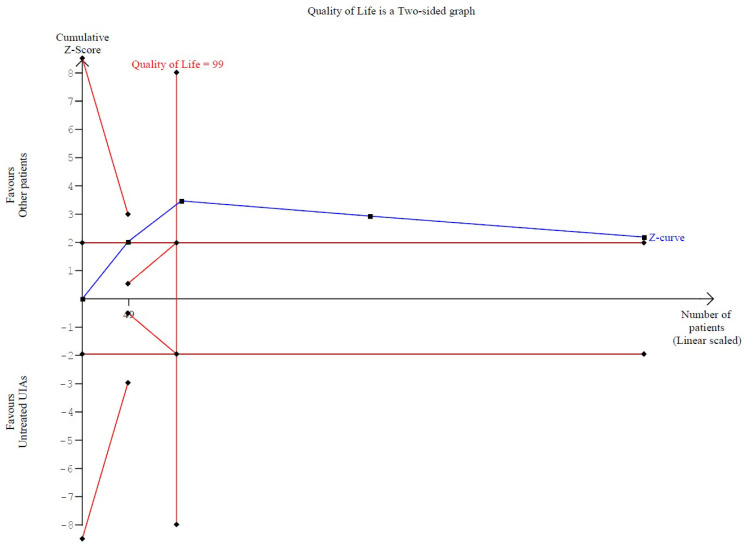
In terms of QoL, there was a statistically significant difference between groups, favoring the control group.

**Figure 10:**
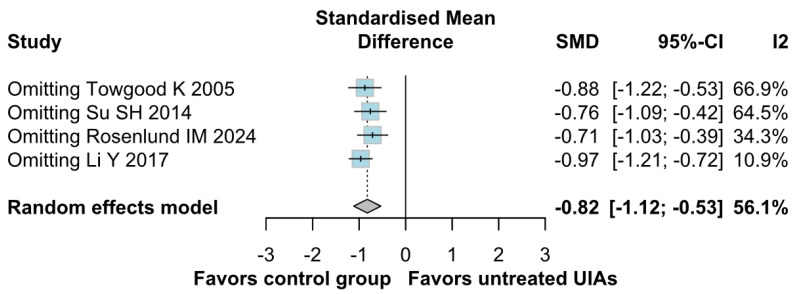
Regarding QoL, the overall effect size remained consistent across all iterations, and the result remained significant in all cases [1,4,31,32].

**Figure 11:**
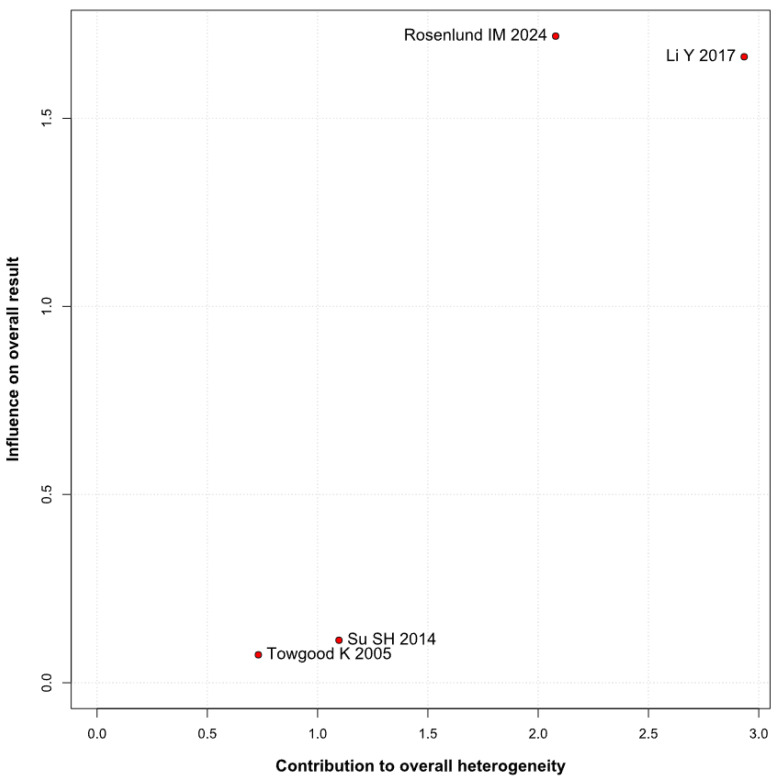
Baujat plot for QoL, showing the contribution of individual studies to overall heterogeneity and their influence on the overall meta-analysis results [1,4,31,32].

A significantly worsened QoL was observed in patients with untreated UIAs (SMD −0.82; 95% CI [−1.12; −0.53]; *p* = 0.01; I^2^ = 56%) compared to the control group (Figure 8). TSA confirmed the significant difference between groups, favoring the control group (Figure 9). A LOO analysis was performed to test the robustness of our results. The overall effect size remained consistent across all iterations, and the result remained significant in all cases (SMD −0.82; 95% CI [−1.12; −0.53]; *p* = 0.01; I^2^ = 56%) (Figure 10). This suggests that no single study has a disproportional influence on the overall outcome. The Baujat plot identified the study by Rosenlund IM 2024 [31] and Li Y 2017 et al. [1] as potentially influential, contributing substantially to the overall heterogeneity, and the result (Figure 11).

### 3.3. Subgroup Analyses

#### 3.3.1. Risk of Bias

##### Anxiety

**Figure 12:**
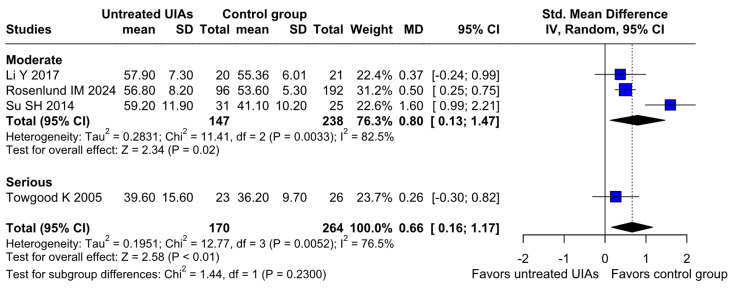
In terms of anxiety, the result did not reach statistical significance between groups [1,4,31,32].

Regarding anxiety, no statistically significant differences were observed between the subgroups (SMD 0.66; 95% CI [0.16; 1.17]; *p* = 0.2300; I^2^ = 76%) (Figure 12).

##### Depression

**Figure 13:**
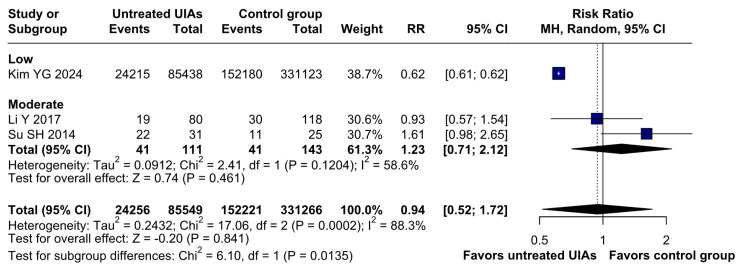
In terms of depression, there was a statistically significant difference between groups [1,3,32].

Regarding depression, a statistically significant difference was observed between the subgroups (RR 0.94; 95% CI [0.52; 1.72]; *p* = 0.0135; I^2^ = 88%) (Figure 13).

##### QoL

**Figure 14:**
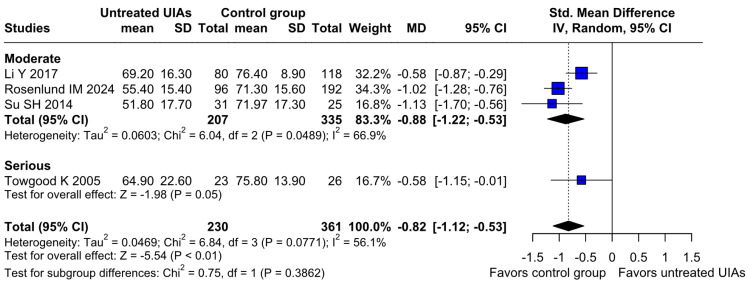
In terms of QoL, the effect did not reach statistical significance between groups [1,4,31,32].

Regarding QoL, there were no significant differences between the subgroups (SMD −0.82; 95% CI [−1.12; −0.53]; *p* = 0.3862; I^2^ = 56%) (Figure 14).

### 3.4. Quality Assessment

**Figure 15:**
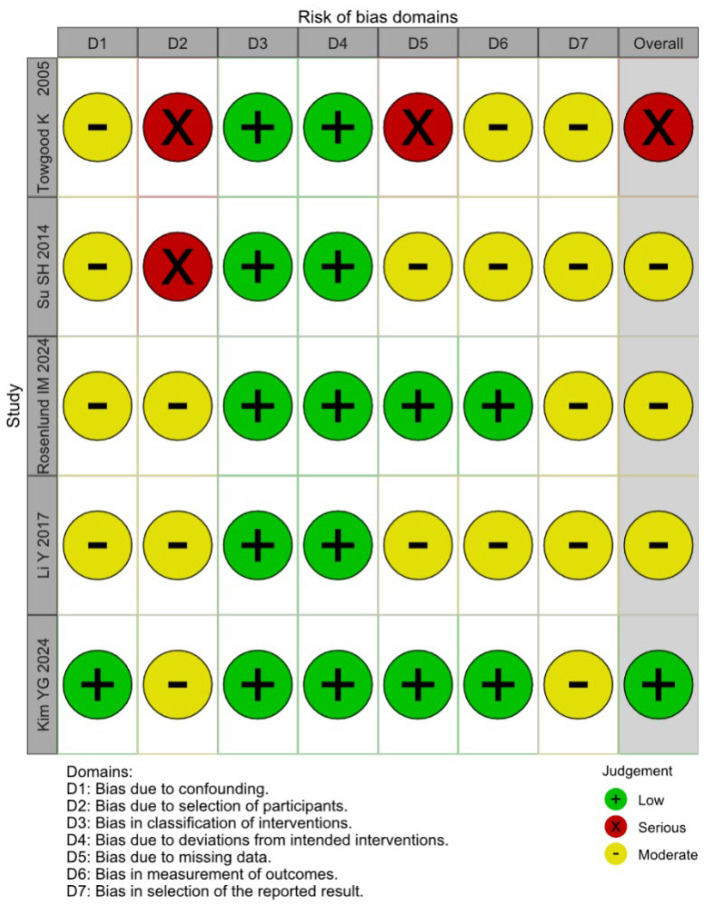
Risk of bias assessment [1,3,4,31,32].

**Figure 16:**
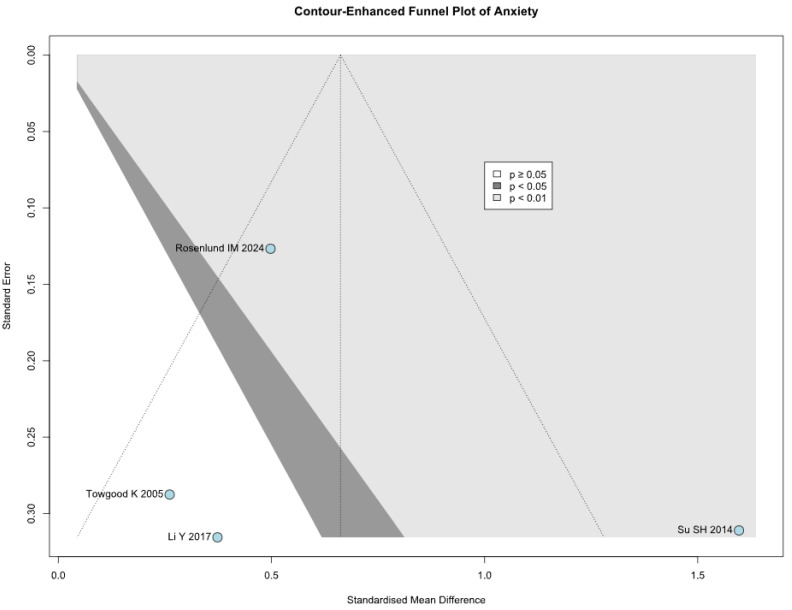
Publication bias evaluation for anxiety, plotting individual study weights against point estimates [1,4,31,32].

**Figure 17:**
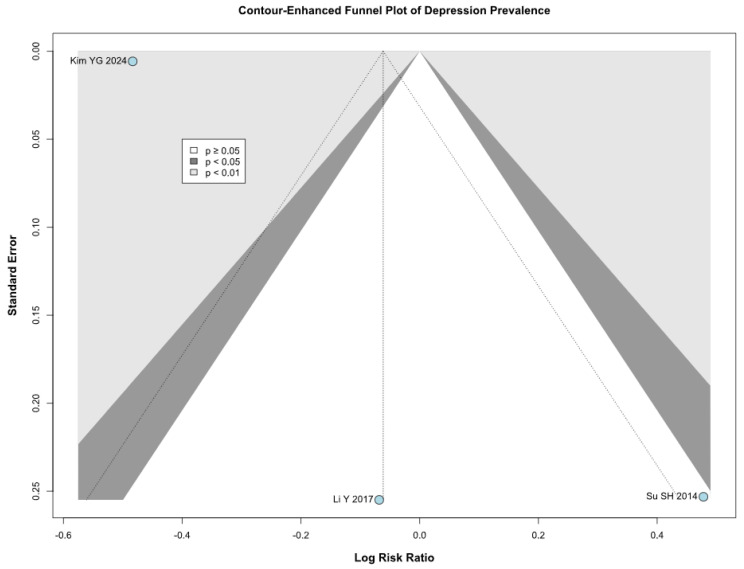
Publication bias evaluation for depression, plotting individual study weights against point estimates [1,3,32].

**Figure 18:**
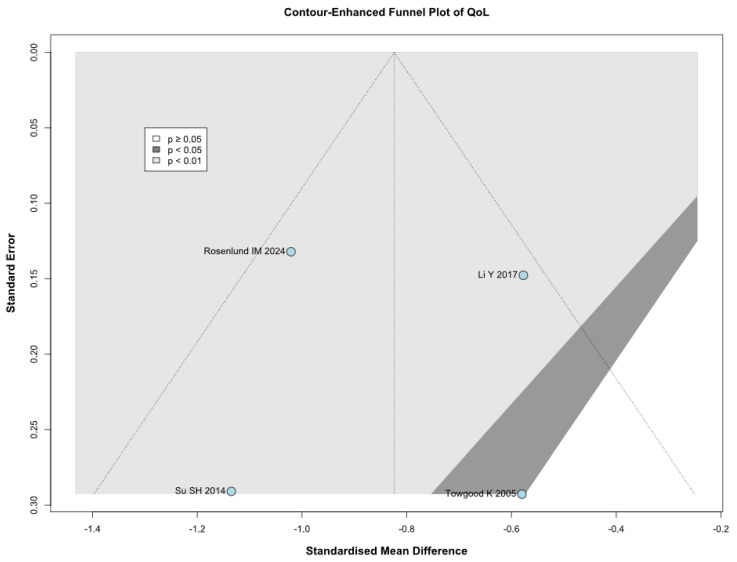
Publication bias evaluation for QoL, plotting individual study weights against point estimates [1,4,31,32].

Among the five included studies, one was judged to have a low risk of bias, three were assessed as having a moderate risk of bias, and one was assessed as having a serious risk based on the ROBINS-I tool. The evaluation of the studies is reported in Figure 15. Among the five included studies, one (Towgood et al., 2005 [4]) was judged to have a serious risk of bias. To evaluate the influence of this study on our findings, we performed sensitivity and subgroup analyses excluding this study. The effect sizes for anxiety, depression, and QoL remained consistent, suggesting that our results are robust despite potential biases. The most common sources of bias were confounding and selection bias, with three studies judged to have a moderate risk of bias and one having a serious risk of bias in these domains. Publication bias was evaluated using funnel-plot analysis, plotting individual study weights against point estimates (Figure 16, Figure 17 and Figure 18). The funnel plots did show asymmetry, but given the small number of included studies, visual interpretation is limited.

## 4. Discussion

In this systematic review and meta-analysis of five studies and 417,152 patients, we compared mental health and QoL outcomes between patients with untreated UIAs and the control group. The main findings from the pooled analyses were as follows: (1) in terms of depression, there were no significant differences between patients with untreated UIAs and the control group; (2) however, patients with untreated UIAs may experience a significantly higher anxiety and lower QoL compared to the control group. Our review highlights the lack of comprehensive data on the psychological outcomes of patients diagnosed with untreated UIAs. This meta-analysis provides an in-depth evaluation of psychological outcomes, specifically anxiety and depression, as well as QoL outcomes associated with untreated UIAs, revealing both neutral and adverse effects. These findings enhance our understanding of the nuanced relationship between untreated UIA diagnoses and their broader psychosocial impacts.

The meta-analysis revealed statistically significant differences in anxiety levels between individuals with untreated UIAs and control groups across four studies [1,4,31,32]. This finding aligns with previous research suggesting that UIAs do universally exacerbate anxiety, particularly over longer durations [3,31]. Depression outcomes across three studies (85,549 intervention and 331,266 control patients), showed no significant differences between groups (*p* = 0.92). An interesting theoretical explanation is that anxiety—characterized by fear of future events—may be more prominent in patients with untreated UIA than depression, which often relates to past experiences. This aligns with the nature of living with a perceived “time bomb”. The Kim YG 2024 study [3], comprising over 400,000 patients, had a considerable impact on the pooled estimates. Although a random-effects model was used, we acknowledge that its large weight may influence the overall results [3]. These observations are consistent with reports suggesting that some patients adapt to the psychological burden of UIAs, leading to minimal long-term distress [1,32]. However, Su et al. reported significantly higher rates of anxiety (84%) and depression (71%) among patients with UIA one year post-diagnosis compared to 16% and 44%, respectively, in the general population [32]. Similarly, Lemos et al. observed that 27% of individuals with UIAs experienced anxiety, but minimal depression was reported [33].

In contrast to the relatively neutral effect observed for depression, QoL measures showed a significant difference favoring control groups. Across four studies (230 intervention and 361 control patients), individuals with untreated UIAs reported lower QoL scores (*p* = 0.03), particularly in mental health domains [1,4,31,32]. These findings are consistent with previous research highlighting the mental distress and reduced well-being associated with living with an untreated UIA, particularly due to the perceived threat of rupture [1,31]. A notable reduction in QoL was also observed in physical functioning, body pain, and mental health domains among untreated patients compared to treated individuals without complications [4,15,34]. While Lemos et al. found no significant impact on QoL three years post-diagnosis compared to the general population [33], Buijs et al. reported a decline in QoL four years after diagnosis without notable changes in mood [34]. Li et al. similarly documented a decrease in QoL without mood alterations, further observing better QoL outcomes for patients diagnosed five years prior compared to those diagnosed one year earlier [1]. Additionally, Su et al. observed an initial decline in QoL, which normalized after five years, with no discernible differences in anxiety, depression, or QoL compared to the general population [32]. Van der Schaaf et al. also reported a decline in QoL, particularly in psychosocial aspects, over an average follow-up of 3.75 years but found no significant increase in anxiety or depression levels [35].

A prior meta-analysis highlighted the psychological impact of unruptured UIAs but differs in focus and methodology [19]. Our meta-analysis emphasizes direct comparisons between patients with untreated UIA and controls, finding no significant differences in depression (OR: 0.95, *p* = 0.92) but reporting significantly higher anxiety (*p* = 0.11) and poorer QoL in patients with untreated UIAs (MD: −8.47, *p* = 0.03). High heterogeneity is a shared challenge addressed using subgroup analyses, LOO, and Baujat plot sensitivity analyses. The meta-analysis by Ignacio et al. estimates pooled prevalence rates of anxiety (28%) and depression (21%) in patients with UIA, with no significant differences between treated and untreated groups [19]. However, while Ignacio et al. [19] reported on the incidence, our analysis provides comparative outcome measures with statistical rigor, including TSA, specifically focused on untreated cases. Moreover, Ignacio et al. focused on prevalence, treatment gaps, and the need for standardized assessment tools, complementing our findings and underscoring shared methodological limitations [19]. While both studies agree on the psychological burden of UIAs and the lack of significant treatment effects, our analysis uniquely quantifies QoL and anxiety outcomes, thereby significantly expanding the current understanding of the physiological and behavioral impact of untreated UIAs.

Furthermore, cognitive outcomes are an important area of concern. Su et al. identified mild cognitive impairment in 97% of patients with untreated UIA over a five-year follow-up period, in stark contrast to preserved cognitive function observed at one year [32]. This underscores the need for long-term monitoring, as untreated UIAs may progressively impact cognitive domains. Towgood et al. corroborated this finding, highlighting subtle but meaningful impairments in memory, attention, and executive functioning among patients with UIA [4]. Together, these findings emphasize the need to consider both QoL and cognitive domains in the holistic management of UIAs. The findings of this meta-analysis underscore the necessity for future studies to employ standardized methodologies to reduce heterogeneity and bias. Given the emerging evidence of cognitive impairment in patients with untreated UIAs, future studies should include standardized neuropsychological assessments as a core outcome alongside measures of anxiety and QoL. This would enable a more comprehensive understanding of the long-term psychosocial and cognitive burden of living with an untreated aneurysm. Longitudinal research that includes diverse populations is essential to better understand the psychological, QoL, and cognitive impacts of untreated UIAs. Interventions such as tailored psychological support, cognitive rehabilitation programs, and proactive management of risk factors like hypertension could help mitigate adverse outcomes. Towgood et al. emphasis on cognitive impairments also highlights the importance of integrating neuropsychological assessments into UIA management strategies [4].

Our study has limitations. The included research showed significant heterogeneity in study design, participant demographics, and follow-up durations, limiting the generalizability of findings. The observed heterogeneity is likely from methodological differences, such as the use of diverse scales (e.g., SF-36, EQ-5D, and HADS) and varying population characteristics including age, geography, and follow-up duration. Although some studies provided data up to five years post-diagnosis, the longer-term impacts on QoL and cognitive outcomes still need to be explored [32]. Additionally, the observational and retrospective nature of many studies introduces a risk of bias, particularly for self-reported measures such as QoL and anxiety [4]. Population representation was another concern, as many studies focused on specific geographic or demographic groups, restricting broader applicability. Finally, uncontrolled psychosocial factors such as socioeconomic status, pre-existing mental health conditions, and healthcare access likely confounded psychological outcome assessments [31]. Although one included study exhibited a serious risk of bias and three others were rated as moderate risk, sensitivity analyses excluding the higher-risk study did not alter the pooled effect sizes. This suggests that the primary outcomes are relatively robust to study-level bias. We attempted to address these limitations with LOO and Baujat plot sensitivity analyses, subgroup analyses based on the risk of bias, and TSA.

## 5. Conclusions

This meta-analysis, including 417,152 patients, suggests a possible association between untreated UIAs and higher anxiety levels and reduced QoL compared to the control group. However, no statistically significant differences were found regarding depression. Future studies with larger, longitudinal cohorts and standardized assessments are warranted to confirm these associations and explore underlying mechanisms.

## Figures and Tables

**Table 1:** Baseline characteristics.

Study	Design	No. Patients	Follow-Up (Years)	Age *	Female, %	Smoker, %	Alcohol Drinker, %	HTN, %	Aneurysmal Size, % #
Li Y 2017 [1]	Retrospective comparative study	P: 80 C: 118	NA	P: 55.8 C1: 58.7 C2: 56.2	P: 70 C1: 79.5 C2: 64.9	P: 28.8 C1: 11.4 C2: 20.3	P: 30 C1: 18.2 C2: 16.2	P: 38.8 C1: 52.3 C2: 48.6	P: [1]-75, [2]-6.3, [3]-18.7 C1: [1]-68.2, [2]-18.2, [3]-13.6 C2: [1]-51.3, [2]-23.0, [3]-25.7
Rosenlund IM 2024 [31]	Retrospective comparative study	P: 96 C: 192	5	P: 65.2 C1: 65.2 C2: 65.2	P: 59 C1: 59 C2: 59	P: 19.2 C1: 13.1 C2: 15.3	P: 11.6 C1: 8.4 C2: 4.8	P: 39.1 C1: 31.4 C2: 29.4	P: 3.1 C: 3.6
Su SH 2014 [32]	Retrospective comparative study	P: 31 C: 25	5	P: 48.1 ± 5.7 C: 49.5 ± 6.6	P: 61 C: 64	P: 39 C: 60	P: 35 C: 44	P: 42 C: 64	P: [1]-83, [2]-11, [3]-6 C: NA
Kim YG 2024 [3]	Retrospective comparative study	P: 85,438 C: 331,123	10	P: 56.41 C: 56.69	P: 49.25 C: 50.56	P: 16.5 C: 15.3	P: 24.9 C: 23.3	P: 47.1 C: 47	NA
Towgood K 2005 [4]	Prospective comparative study	P: 23 C: 26	NA	P: 50.22 C: 48.73	P: 70 C: 58	P: 36 C: 62	P: 41 C: 23	P: 55 C: 35	P: [4]-52, [5]-17, [6]-9, [7]-22 C: [4]-42, [5]-31, [6]-19, [7]-8

* Mean in years; C1—control group 1; C2—control group 2; P—population; HTN—hypertension; # aneurysmal size; [1] < 7 mm, [2] 7–10 mm, [3] > 10 mm, [4] 2–5 mm, [5] 6–9 mm, [6] 10–14 mm, [7] >15 mm.

## Data Availability

Not applicable.

## Supplementary Materials

The following supporting information can be downloaded at: https://www.mdpi.com/article/10.3390/brainsci15070764/s1, File S1: PRISMA 2020 Checklist.
